# Supplementary material for: Mutations in PCYT2 disrupt etherlipid biosynthesis and cause a complex hereditary spastic paraplegia
Source: Brain. 2019 Oct 22;142(11):3382–97. doi: 10.1093/brain/awz291 (PMC6821184; doi:10.1093/brain/awz291)
Supplement: awz291_Supplementary_Data [file awz291_supplementary_data.zip › awz291-Suppl_data/Supplementary_Data4.zip!/Supplementary information 4 - plasma lipidomics.zip!/Plasma lipidomics/Control vs Patient3b/Statistical plots and data/LPC[O] species/Volcano plot_alternative.pdf]

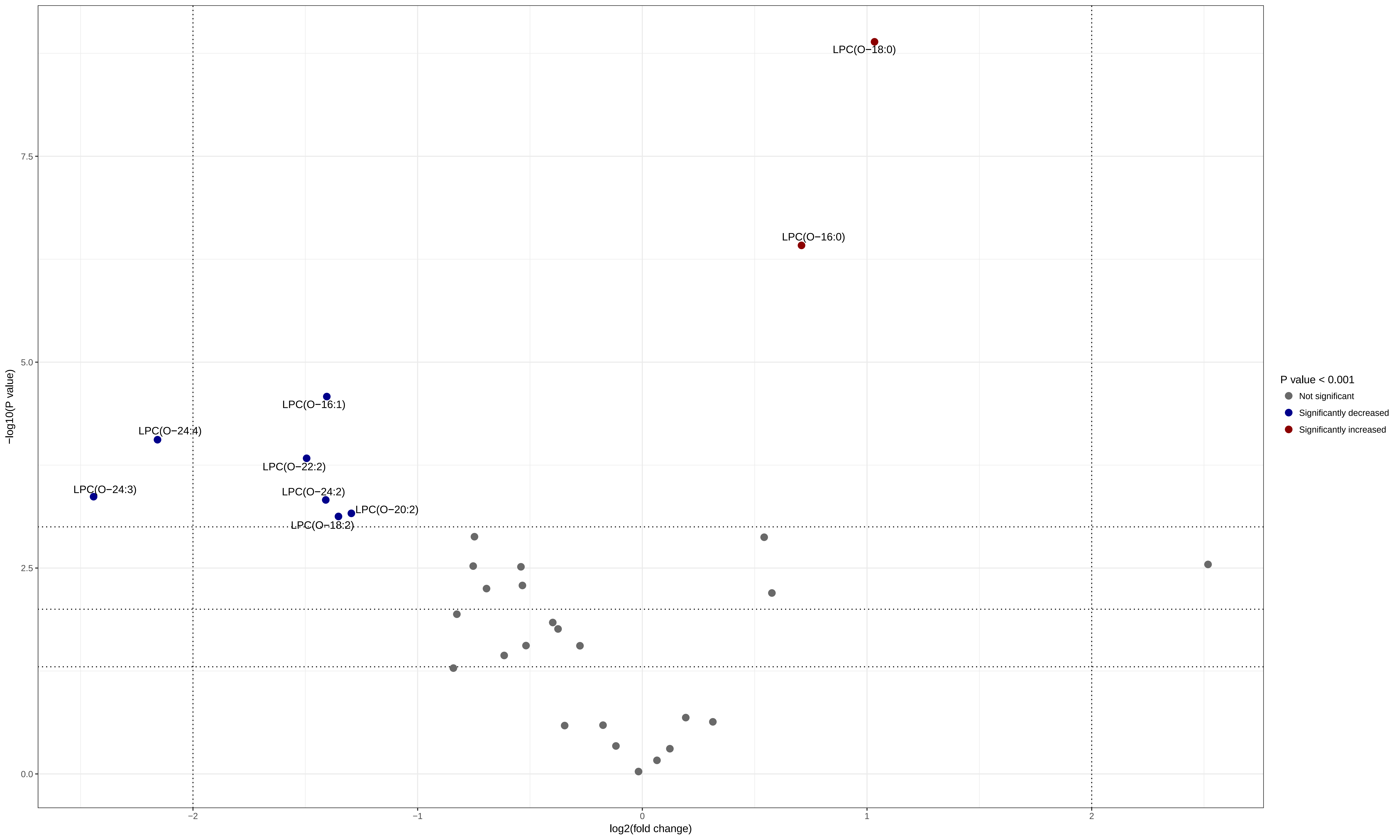
LPC(O−18:0)
LPC(O−16:0)
LPC(O−16:1)
LPC(O−24:4)
LPC(O−22:2)
LPC(O−24:3)
LPC(O−24:2)
LPC(O−20:2)
LPC(O−18:2)
7.5
5.0
2.5
0.0
−log10(P value)
−2
−1
0
1
2
log2(fold change)
P value < 0.001
Not significant
Significantly decreased
Significantly increased
